# Case report: Magnetic resonance imaging-based three-dimensional printing for reconstruction of complex cloacal malformations

**DOI:** 10.3389/fped.2023.1103401

**Published:** 2023-03-07

**Authors:** Suiin Gang, Sang Hoon Song, Jaeyoung Kwon, Hyunhee Kwon, Suhyeon Ha, Jueun Park, Namkug Kim, Hee Mang Yoon, Jung-Man Namgoong

**Affiliations:** ^1^ Department of Pediatric Surgery, Asan Medical Center, Seoul, Republic of Korea; ^2^ Department of Urology, University of Ulsan College of Medicine, Asan Medical Center, Seoul, Republic of Korea; ^3^ Anymedi Inc., Gyeonggi-do, Republic of Korea; ^4^ Department of Radiology, Convergence Medicine, University of Ulsan College of Medicine, Asan Medical Center, Seoul, Republic of Korea; ^5^ Department of Radiology and Research Institute of Radiology, University of Ulsan College of Medicine, Asan Medical Center, Seoul, Republic of Korea

**Keywords:** cloaca, reconstruction, three-dimensional printing (3D printing), magnetic resonance imaging, contrast

## Abstract

**Background:**

Surgical reconstruction of the urinary tract, anus, and vagina is the definitive treatment for cloacal malformation. However, this procedure may be technically challenging in patients with a long common channel (>3 cm), because further reconstructive procedures, such as vaginal replacement or vaginal switch maneuver, may be required. Thus, accurate determination of spatial anatomy is essential during surgical planning. Three-dimensional (3D) reconstruction using rotational fluoroscopy, computed tomography (CT), and magnetic resonance imaging (MRI) has recently been reported to help in determining the relationship between the rectum, vagina, and bladder, and provides a more accurate measurement of the channel length compared to conventional cloacography. MRI-based 3D reconstruction provides substantial information regarding soft tissue structures around the cloaca, including the pelvic floor musculature and anus.

**Case:**

A 2-year-old girl with cloacal malformation required reconstructive surgery. Colostomy and cystostomy had been performed on the first day of her life. Preoperative loopogram revealed a cloaca with a long common channel (35 mm) and short urethra (9 mm), single vaginal opening in the bladder neck, and the colon anterior to the vagina with a fistula at the vaginal neck. Because the vagina was too short to be pulled through, 3D printing based on MRI was performed to visualize structural relationships prior to surgical correction. Saline was used for cloacal visualization. Furthermore, endoscopy-assisted urogenital mobilization was performed, and vaginal substitution was performed using the rectum. No postoperative complications were observed.

**Conclusions:**

We believe this is the first report of the use of MRI-based 3D imaging and printing, with saline as a contrast agent during surgical planning for correction of cloacal malformation. MRI-based 3D printing is a potentially promising technique for surgical planning of cloacal malformation correction in patients with a long common channel, as it provides detailed information about the surrounding soft tissue structures without exposure to radiation or contrasting agents.

## 1. Introduction

Cloacal malformation is rare and one of the most complex forms of female urogenital tract malformation accounting for one in every 50,000 live births (1). Accurate determination of the cloacal anatomy is crucial for successful corrective surgery. The length of both the common channel and urethra, anatomy of the upper genital tract, and the location of fistulae, specifically rectal fistula, and the true rectum must be considered while planning the surgery (2). Furthermore, determination of relative position of the rectum in the pelvis and its relationship to the pubococcygeal line is vital. Moreover, obtaining accurate information on spatial anatomy is important when deciding on the most appropriate method of surgical reconstruction in patients with a long common channel and a short urethra. The distance and three-dimensional relationship (angle) between the urethra and common channel is a decisive factor in determining the most convenient surgical reconstruction method (3–6). Furthermore, the pelvic cavity may be narrow in those patients (male pelvis), and they may have genital malformations requiring further reconstruction of the vagina. The need for urogenital separation and a short vaginal length has been posited as predictive factors for vaginal replacement (7). Three-D reconstruction and printing has been reported to provide accurate visualization and quantification of the size and spatial relationship of each cloacal structure in the pelvis. Most previous studies of 3D reconstruction were based on images obtained by computed tomography (CT) and fluoroscopy (8–10). In a previous study, magnetic resonance image (MRI)-based three-dimensional (3D) cloacagram was performed using low-osmolarity iodinated contrast and gadolinium as contrast agents (11). Another study reported that 3D reconstruction and printing during surgical planning procures a more accurate length measurement of the common channel and urethra compared to 2D fluoroscopy (6). Herein, we demonstrate the use of MRI-based 3D printing, using saline as a contrast agent, during surgical planning of cloacal malformation correction leading to a successful postoperative outcome.

## 2. Case description

A two-year-old girl was admitted to the pediatric surgery department at the Asan Medical Center for a cloacal anomaly correction. The patient was born at a gestational age of 32 weeks *via* a cesarean section and underwent loop colostomy and cystostomy formation on the first day of life. Hydrocolpos was relieved after cystostomy because the retension of the unexcreted urine into the vagina is resolved. The patient later developed a high-grade vesicoureteral reflux affecting both ureters (grade IV on the right and grade V on the left) with recurrent urinary tract infections.

## 3. Diagnostic assessment

**Figure 1:**
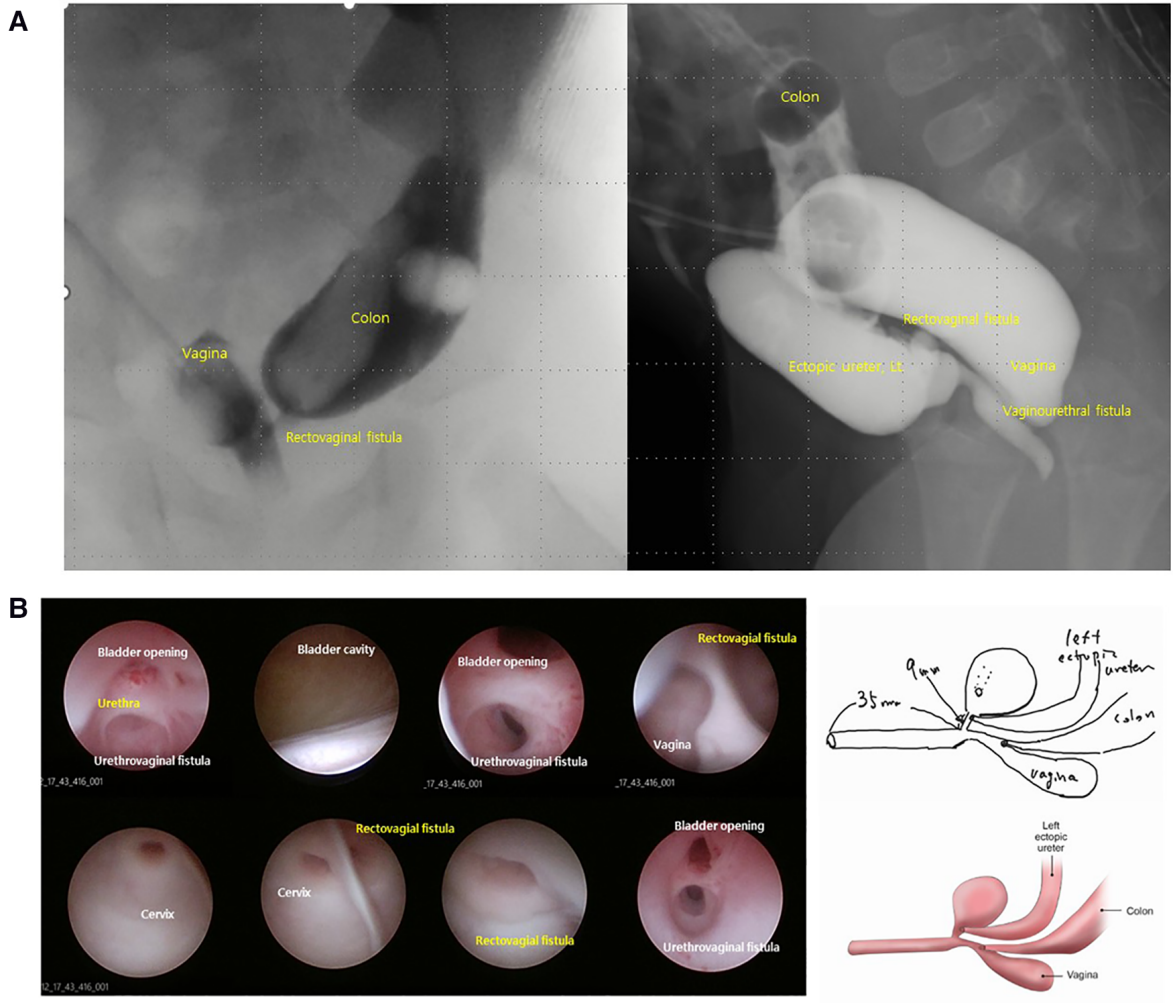
Images of distal loopogram (**A**) and endoscopy (**B**). Distal loopogram revealing the fistula location and spatial relationship between each cloacal structure. Loopography provides limited information on the exact size or location of individual structures. Similarly, endoscopy alone is unable to accurately determine the size or position of individual structures. This schematic based on endoscopic findings demonstrates the limitations of endoscopy.

Preoperative colon study (distal loopogram) showed a vaginourethral fistula (between the distal anterior vaginal wall and posterior wall of the mid urethra), rectovaginal fistula (between the upper rectum and the left lateral wall of vagina), and a dilated, ectopic left ureter below the bladder neck (Figure 1A).

Endoscopy revealed a common channel length of 35 mm and a short urethra with a length of 9 mm (Figure 1B). A possible fistula was observed between the vagina and urethra. However, it was difficult to accurately assess the size of each lumen and the spatial relationship between the structures based on endoscopic findings alone.

**Figure 2:**
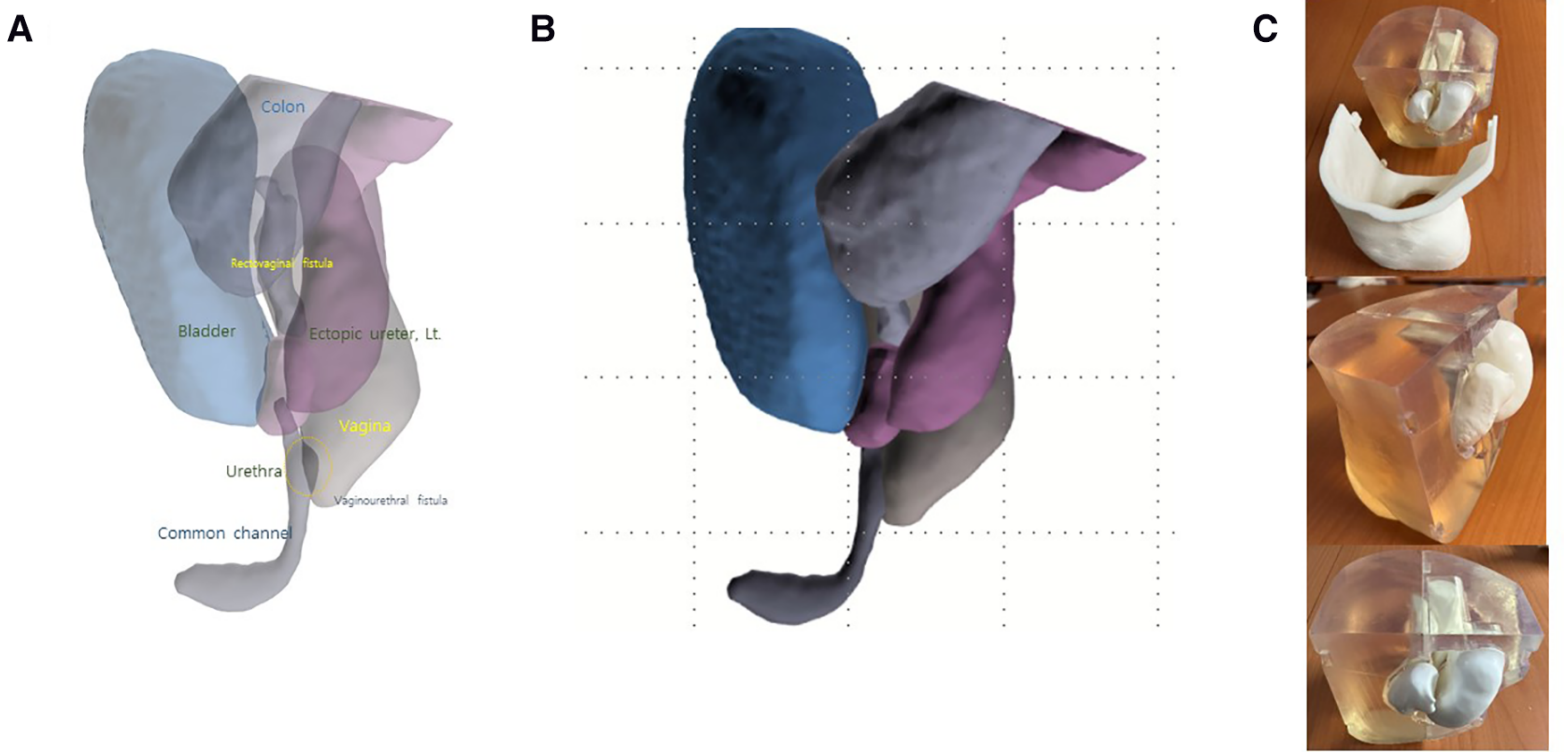
Three-dimensional (3D) reconstruction images (**A**,**B**) and printing (**C**) of the cloaca in the present case. Each structure (**A**,**B**) and their spatial relationship in the pelvic cavity (**C**) can be identified using 3D reconstruction. The length measured from 3D reconstruction was compared that measured by endoscopy. Urogenital separation was performed based on common channel and urethral lengths determined from 3D reconstruction images. 3D reconstruction images suggested the requirement for vaginal reconstruction. Thus, vaginal substitution using a dilated rectum was performed.

MRI cloacagram was performed preoperatively according to our own protocol using saline as a contrast agent. Prior to examination, saline was infused *via* Foley catheters into the colostomy, cystostomy, and common channel (primitive urogenital sinus). MRI scan was performed after dilatation of the cloacal system with saline using 3.0 T MRI (Ingenia Elition, Philips Medical Systems, Best, Netherlands). The colostomy and cystostomy were manually occluded during MRI to allow visualization of the dilated cloacal lumens, fistulae, and associated spaces. Three-D reconstruction was performed to determine the size of the pelvic structures based on the outer wall of each luminal organ (Figure 2). The length of each structure measured using 3D reconstruction images was comparable to endoscopic measurements. Thus, we decided to perform urogenital separation due to the presence of a short urethra (9 mm), based on 3D printing of reconstructed images.

## 4. 3D Printing

We used relatively inexpensive polyacetic acid (PLA) and took fused deposition modeling (FDM) method to reduce production cost for printing internal organs. The body (case) was 3D printed with stereolithography (SLA) method using clear resin to secure transparency while holding organs. Internal organs including ureter, urethra, colon, bladder, and vagina were 3D printed by FDM method. FDM is a method of making a shape by melting a material at a temperature above its melting point, forming a layer while drawing a line with a certain thickness, and stacking them up. SLA is a method of irradiating and curing photopolymer resin with a laser to create and stack layers on a printing bed. Ultimaker S5 (Ultimaker BV, the Netherlands) of FDM type and Form3 (FormLabs©, Cambridge, MA, United States) of SLA type were used for printing.

## 5. Surgical treatment

**Figure 3:**
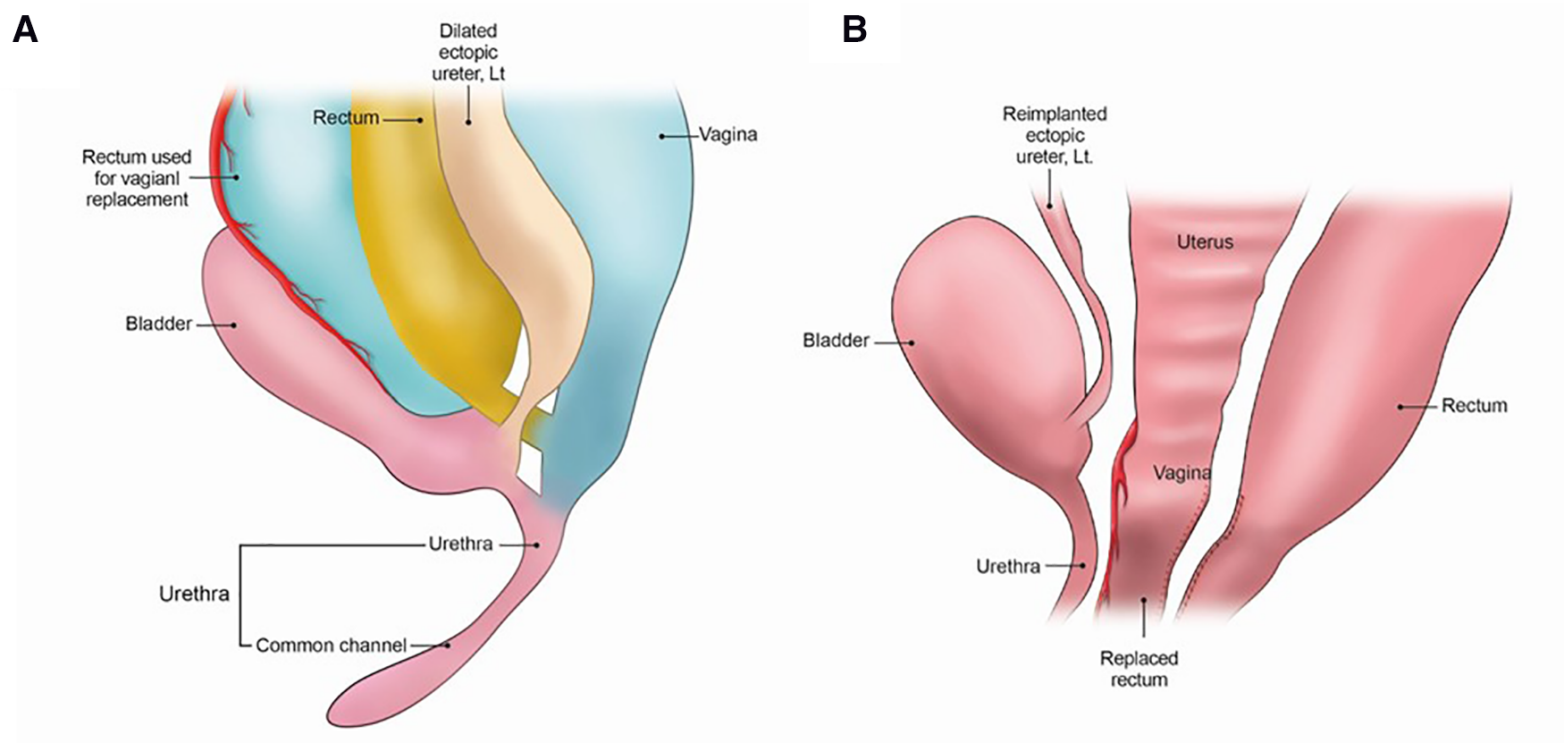
Selection of laparoscopic-assisted US with vaginal replacement as the most appropriate surgical approach. Mobilization was performed via a laparoscopic approach. Urogenital separation (US) was performed due to the presence of a short urethra (9 mm in length). Vaginal replacement was performed using a dilated rectum after distal resection of the vagina as it was extremely difficult to fully pull through the dilated vagina. (**A**) Before operation (**B**) Post-operative status.

She underwent laparoscopy-assisted anorectal pull-through procedure. We decided to perform vaginal reconstruction of the short and dilated vagina using the dilated rectum and considering the narrow pelvic space (Figure 3). Mobilization of intraabdominal compartment was performed *via* a laparoscopic approach. As the dilated vagina was not completely pulled through, part of the distal vagina was excised, and vaginal replacement was performed by longitudinally excising the dilated rectum immediately superior to the fistula. Left ectopic ureter was excised along with vaginal dissection and was tapered and reimplanted into the right posterior wall of the bladder (Cohen reimplantation procedure). Right ureter was also reimplanted by Politano-Leadbetter technique to prevent reflux. Vaginoplasty was performed using perineal skin flap. The postoperative course was uneventful, and the patient was discharged with no acute complications. Moreover, Hegar dilatation of the anus and vagina and clean intermittent catheterization were performed on an outpatient basis. Finally, a clinical follow-up confirmed spontaneous urination without intermittent catheterization.

## 6. Discussion

Three-D printing was first developed in the 1980 s and substantially impacted the advancement of medical technology. 3D printing was rapidly incorporated into existing medical imaging, including CT and MRI, to facilitate 3D image reconstruction and visualization of deep anatomical structures. After it became widely available, 3D printing technology is now used to produce patient-specific organ replicas allowing surgeons to meticulously plan preoperative surgical procedures using accurate three-dimensional reconstructions. Several studies have highlighted the importance of 3D cloacagram and 3D printing of cloacal reconstructions (6, 10). This approach has facilitated the accurate determination of the spatial anatomy of the cloacal systems and has assisted in the selection appropriate surgical procedures (12). Three-D printing has also contributed to the education of young surgeons and scientific research fellows.

There are currently several options for cloacal visualization (13). Endoscopy has traditionally been considered the standard method of visualizing the detailed anatomy of the urogenital tracts, fistulae, and related structures. However, the exact spatial relationship between individual structures can be difficult to determine using endoscopy alone. Fluoroscopy produces a 2D image and enables visualization of fistulae and related structures through the administration of contrast. Conversely, fluoroscopy has limited application in providing information on three-dimensional structures. Several recent publications have demonstrated the use of CT-based 3D reconstructions in understanding 3D structures. MRI provides greater soft tissue definition than fluoroscopy and CT. Also, it can provide anatomical data on pelvic musculature and hollow visceral structures related to cloacae. MRI can also delineate non-opacified lumens, such as obstructed vaginal cavities (11). However, MRI has limited utility in the direct visualization of fistulae that are not dilated with fluid, but limitation can be overcome by performing MRI cloacagram with contrast injection *via* cystostomy. Preoperative MRI cloacagram, a T2 imaging technique comparable to magnetic resonance cholangiopancreatography, was performed using water as a contrast agent in the present case. This technique provides both MRI images and cloacagram, while eliminating radiation exposure and nephrotoxicity. We used 3D reconstruction to visualize three-dimensional structures and measure the exact length of individual pelvic structures during surgical planning. Halleran et al. proposed that 3D cloacagram should be performed in all patients prior to cloacal malformation reconstruction (6). Gasior et al. reported that 3D reconstruction images are superior to conventional endoscopy in measuring the length of the common channel and urethra (12). The difference between the length measured using 3D reconstruction images and endoscopy may be greater in patients with a long common channel due to a greater angle between the common channel and the urethra. But we can measure the length with greater accuracy when using 3D reconstruction (6). Three-D reconstruction images were printed to provide accurate visualization of the three-dimensional structure of the cloacal system prior to surgery and to devise a meticulous surgical plan, considering the urogenital separation and vaginal substitution in the present case.

Establishing an accurate plan is important in pediatric reconstruction surgery, which can permanently influence the quality of life of the child. Cloaca reconstruction requires multi-stage operations from birth to childhood, and the outcome of each surgery affects the plan for the next surgery and the final reconstruction result. Through the MRI cloacagram using water as a contrast agent, we were able to obtain information not only about the lumen but also the three-dimensional structure of the structure itself and the surrounding pelvic cavity without any risk to the patient. 3D printing has helped simulate various surgical plans, including vaginal substitution, before definite surgery. By performing accurate reconstruction, we were able to efficiently reconstruct the urogenital system, and so far, both the patient and their caregivers are living stably without additional reconstructive surgery.

We believe this to be the first case of cloacal malformation correction where a surgical plan was established using 3D printing based on MRI cloacagram. We expect that 3D image based on MRI cloacagram using saline as contrast agent may eventually replace endoscopic examinations as it provides more accurate anatomical information during surgical planning for children with complicated cloaca. By substituting the information given by cystoscopy, it was possible to save time and cost incurred by performing cystoscopy. The cost required for 3D printing has recently become much cheaper, and with the technique used in this article, it can be produced within a week at a cost of less than 100 United States dollars. Additionally, MRI-based 3D reconstruction may replace existing CT-based 3D image as it provides better information on surrounding soft tissues with no risk of exposure to contrast agents or radiation. The advantage of this technique is that it can replace cystoscopy and also provide more intuitive information to the surgeon by grafting the cloacagram to MRI and adding 3D printing technology to it. The additional advantage of reducing two anesthesia to one, especially in young patients, can also be considered.

The 3D image based on the MRI cloacagram itself would provide sufficient information to proficient surgeons in planning surgery. Moreover, 3D printing can provide more intuitive information to fellows or surgeons with limited experience. Through the process of manipulating the printing, by simulating the surgery process in advance, surgeons can directly check the results of the surgery with their own eyes, so they can set up a more accurate surgery plan. As such, 3D printing has the advantage of making an additional contribution to the learning of surgeons in the growing process.

## Data Availability

The original contributions presented in the study are included in the article/Supplementary Material, further inquiries can be directed to the corresponding author.
